# Trade-offs between driving nodes and time-to-control in complex networks

**DOI:** 10.1038/srep39978

**Published:** 2017-01-05

**Authors:** Sérgio Pequito, Victor M. Preciado, Albert-László Barabási, George J. Pappas

**Affiliations:** 1 Department of Electrical and Systems Engineering, School of Engineering and Applied Science, University of Pennsylvania, Philadelphia, Pennsylvania 19104, USA; 2 Center for Complex Network Research and Departments of Physics, Computer Science and Biology, Northeastern University, Boston, Massachusetts 02115, USA; 3 Center for Cancer Systems Biology, Dana-Farber Cancer Institute, Boston, Massachusetts 02115, USA; 4 Department of Medicine, Brighamand Women’s Hospital, Harvard Medical School, Boston, Massachusetts 02115, USA

## Abstract

Recent advances in control theory provide us with efficient tools to determine the minimum number of driving (or driven) nodes to steer a complex network towards a desired state. Furthermore, we often need to do it within a given time window, so it is of practical importance to understand the trade-offs between the minimum number of driving/driven nodes and the minimum time required to reach a desired state. Therefore, we introduce the notion of *actuation spectrum* to capture such trade-offs, which we used to find that in many complex networks only a small fraction of driving (or driven) nodes is required to steer the network to a desired state within a relatively small time window. Furthermore, our empirical studies reveal that, even though synthetic network models are designed to present structural properties similar to those observed in real networks, their actuation spectra can be dramatically different. Thus, it supports the need to develop new synthetic network models able to replicate controllability properties of real-world networks.

In recent years, a powerful arsenal of tools has been developed to control the dynamics of complex networks, integrating knowledge from the fields of control theory, network science, and statistical physics^1^. In this direction, control theory equips us with powerful mathematical notions, such as *controllability* and *controllability subspace*^2^,^3^, to determine the set of dynamic states that are achievable (in finite time) by carefully choosing external driving signals. Even though most of these tools require full access to the network dynamics, in many practical scenarios, either the dynamics leads to a *ill-posed* controllability problem^4^ or only the topology of the dynamic network is available. In this context, it is still possible to analyze network control problems using tools from *structural control theory*. Structural control theory enables us to draw conclusions about controllability properties of *almost all* dynamic networks sharing the same topology using graph-theoretic methods^5^,^6^,^7^,^8^. Using these tools, a collection of interesting network control problems has been recently addressed in the field of network science^1^,^9^,^10^. One of such problems consists of finding the minimum number of driving (or driven) nodes to steer a dynamic network towards a desired state^11^. Using structural controllability, the minimum number of driving^9^,^12^ and driven nodes^13^ can be found when only the topology of the dynamic network is available by solving a maximum bipartite matching problem. Similar problems can also be solved while considering actuation costs^14^,^15^, energy constraints^16^, edge dynamics^17^,^18^, or constraints on the set of controlled states^19^.

Current control tools mainly focus on our ability to steer the network dynamics towards a required state, without any regards to the required control time. Nonetheless, in many biological, social, and technological networks, it is of practical importance to ensure that the networks’ states are steered to a predefined goal within a small time window. In control theory, the *controllability index*^3^ characterizes the minimum time required to steer a dynamic network towards a desired state with a given set of driving/driven nodes. Furthermore, when only the network topology is available, we can use the notion of *structural controllability index*^20^,^21^ from structural control theory. In this work, we use these notions to explore the trade-offs between the time-to-control and the minimum number of driving/driven nodes in a variety of real and synthetic network topologies. To visually capture these trade-offs, we introduce the concept of *actuation spectrum* of a dynamic network, which characterizes the minimum number of driving/driven nodes to control the network for any time-to-control. Therefore, it allows us to characterize our ability to steer the dynamics of a network under time constraints.

From an empirical analysis of the actuation spectra for a wide variety of artificial and synthetic networks, we observe that, in many cases, only a small fraction of driving/driven nodes is required to steer the network to a desired state within a relatively small time window. Our empirical observations also reveal that, even though artificial network models are designed to present structural properties similar to those observed in real networks, real-world networks present, in general, different actuation trade-offs than their artificial counterparts. Therefore, our studies support the need to develop new synthetic network models able to replicate not only structural metrics (such as degree distributions), but also controllability properties of real-world networks.

## Results

Let us model the dynamic evolution of a complex network by the following linear discrete time-invariant system:


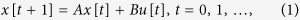


where 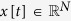 is a vector containing the states of all the nodes in the network at time *t, x*[0] = *x*_0_ is the initial state, and 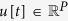 is the value of the *P*-dimensional input signal injected in the network at time *t*. The matrix 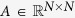 is the state matrix, which captures the dynamic interdependencies among nodes; the matrix 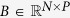 is the input matrix, which identifies those nodes that are actuated by an external input signal. Equation (1) models can be used to model the dynamics of networks, as well as the local linearization of non-linear dynamical^22^. In addition, given *A* and *B*, the *partial controllability matrix* of order *T* is defined as


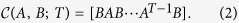


When *T* is equal to *N* (i.e., the dimension of the state space), the matrix 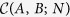 is referred to as the *controllability matrix* of the system^2^. A system is *controllable* if, for every initial condition 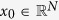, there exists an input signal 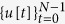 able to steer the system to any arbitrary final state 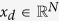 in at most *N* time steps. Kalman’s controllability criterion^2^ states that a system is controllable, if and only if, 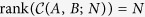.

In many practical settings, we are interested in steering the state of a large-scale complex networks within a time window much shorter than *N*. In this case, we need to modify the definition of controllability to account for the time required to steer a system. In this direction, control theory provides the concept of *controllability index*, which is defined as the minimum value of *T* for which the partial controllability matrix 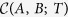 is full rank. Formally, the controllability index is defined as follows:


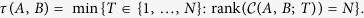


From a dynamic point of view, the controllability index is equal to the minimum number of time steps required to steer the system from *x*_0_ to an arbitrary final state *x*_*d*_. In particular, if the system is controllable and the initial state is the origin (i.e., *x*_0_ = 0), the input signal 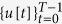 that steers the system to 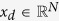 can be explicitly computed as ref. 2


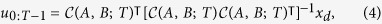


where 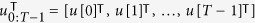 is a vector in 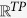 containing a concatenation of the input signal. Notice that, for *T* ≥ *τ*(*A, B*), the matrix inside the brackets in (4) is invertible and *u*_0:*T*−1_ is well-defined. The controllability index can be easily extended continuous-time dynamical systems^3^. Nonetheless, because current technology relies in digital controllers, we focused on discrete-time dynamics (for instance, resulting form the discretization of continuous time dynamics) to obtain a control law that steers the system towards a desired state.

However, in many contexts, it is not possible to exactly retrieve the dynamic interactions among network variables, but we have access to the topology of the network over which the dynamics takes place. In other words, in some cases it is not possible to exactly retrieve the content of the matrices *A* and *B*, but we have access to the location of their nonzero entries (i.e., the location of the edges in the network). In this context, we can use tools from *structural controllability theory* to study controllability properties of almost all networks sharing the same topology. This can be achieved by analyzing graph-theoretic properties of the *system digraph*, which is constructed by associating vertices to both state variables and input signals. The edges of the system digraph are determined by the entries of the matrices in (1). More precisely, if *A*_*ij*_ is non-zero, there exists an edge from the state vertex *x*_*j*_ to *x*_*i*_. Similarly, if *B*_*l*,*m*_ is non-zero, then there exists an edge from the input vertex *u*_*m*_ to the state vertex *x*_*l*_. In particular, the *state digraph* corresponds to the subgraph of the system digraph that contains only state vertices. Remarkably, structural controllability can be assessed by resorting to the notion of an *input cactus*, which is inductively defined as follows: (*i*) a directed path with at least two vertices, where the origin is an input vertex and the remaining are distinct state vertices, is referred to as an *input stem*, and it is an input cactus; and (*ii*) an input cactus connected by an edge to a disjoint cycle containing only state vertices is also an input cactus. A major result in structural controllability theory states that a system is structurally controllable, if and only if, the system digraph contains a disjoint union of input cacti spanning the system digraph^23^,^24^. Additionally, given a state digraph, we can find the minimum number of *driving* nodes (i.e., the minimum number of inputs required to ensure structural controllability) by solving a maximum matching problem^7^,^9^,^12^. More recently, it was shown that the minimum number of *driven* nodes (i.e., the minimum number of state vertices that need to be actuated to ensure structural controllability) can be obtained by solving a minimum weighted maximum matching^13^. Notice that the minimum number of driven nodes is always greater or equal to the minimum number of driving nodes.

In structural control theory, the notion of *structural controllability index*^20^,^21^ is concerned with the trade-off between the number of driving/driven nodes and the time required to steer a structural system to a desired state. This index is defined as follows: Consider the structural matrices 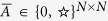 and 
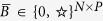
, where the entries are either 0 (i.e., there is no edge between two nodes), or an unknown nonzero entry (i.e., there is an edge between two nodes with an arbitrary weight) denoted by 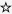. In other words, the matrices 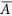 and 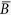 characterize the topology of the system digraph, when the weights can take any arbitrary value. Given a structural state matrix 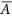 and a structural input matrix 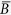, we say that the corresponding structural system is structurally controllable with index *T* if there exists a pair of real matrices (*A, B*) corresponding to a weighted realization of the system digraph such that the controllability index of (*A, B*) is equal to *T*. In other words, we can find a (weighted) network with a system digraph matching the topology described by the pair 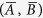 such that it can be controlled in (at least) *T* time steps. This value of *T* is called the *structural controllability index*, which we denote by 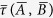. In fact, using functional analysis^6^, almost all weighted networks associated with such system digraph can be controlled in at least *T* time steps. In other words, any random assignment of weights to the edges of the system digraph would result (with high probability) in the same time-to-control.

**Figure 1:**
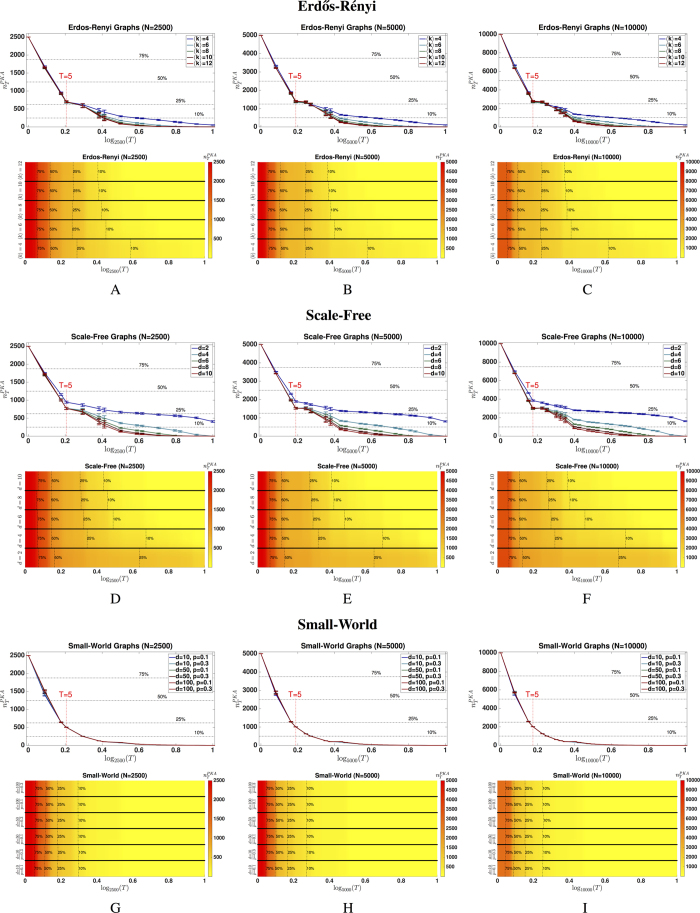
Input Cacti and Proposed Two-Step Approach. In (**A**) we depict a system digraph, and in (**B**) and (**C**) two possible disjoint spanning input cacti. Notice that in B one input cactus has nine state vertices and the other six, whereas in (**C**) one input cactus contains eight state vertices and the other seven. In fact, these are the only two spanning input cacti, so the structural controllability index is equal to eight. Our two-step approach is depicted in (**D**–**F**). First, given the state digraph in (**D**) we consider a partition with at most eight state vertices, leading to two partitions denoted by *P*_1_ and *P*_2_. Secondly, in (**E**) we find the minimum number of driven nodes that correspond to the roots of a disjoint union of state cacti containing all the vertices in each partition. Finally, we just need to assign inputs to the driven nodes associated with nodes 1 and 11 respectively, as depicted in (**F**).

As we illustrate below, the structural controllability index is a powerful tool to understand the minimum number of time steps required to steer a network to a desired state. Furthermore, this index can be described in graph-theoretic terms as follows: a pair of structural matrices 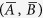 is structurally controllable with index 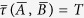, if and only if, the system digraph is spanned by a disjoint union of input cacti, where every input cactus contains at most *T* state nodes (see *SI Text*, section II, Theorem 2). In Fig. 1, we depict a particular system digraph, as well as two different disjoint unions of input cacti, to illustrate the graph-theoretic interpretation of the structural controllability index.

### Actuation Spectrum

**Figure 2:**
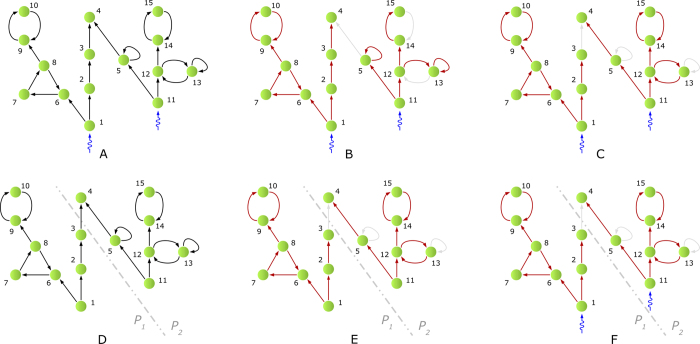
Actuation Spectrum. Figures (**A–C**) depict the actuation spectra of three networks with *N* = 100 nodes using a heat-map with colors ranging from yellow to red, where yellow (respectively, red) corresponds to a low (respectively, high) number of driving nodes (denoted by 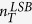) or driven nodes (denoted by 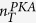) required to control the network in at least *T* time steps (represented in the *x*-axis using the scale log_100_(*T*)). Notice that the highest number of driving/driven nodes (darkest red) is required when *T* = 1 (or, log_100_(*T*) = 0), since we need to actuate all the nodes to drive the network state in a single time step (i.e., 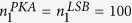). Similarly, the lowest number of driving/driven nodes (brightest yellow) is achieved in the absence of time constraints (i.e., *T* = 100 or log_100_(*T*) = 1). In addition, we mark by vertical dashed lines the values of log_*N*_ (*T*) for which the number of required driving/driven nodes corresponds to 25%, 50% and 75% of the network size *N*. The three networks under consideration exhibit qualitatively different decays in the number of driving/driven nodes as *T* increases. In particular, the faster the decay in the actuation spectrum, the easier it is to control the network in a short time window. We, therefore, say that a network is ‘agile’ if its actuation spectrum decays fast as a function of *T*. In this sense, the Type-III network in (**C**) is the most ‘agile’, while the Type-I in A is the least ‘agile’. Notice how the actuation spectrum of an ‘agile’ network decays fast to the yellow level in the heat-map, or, equivalently, the vertical dashed lines are shifted to the left.

**Figure 3:**
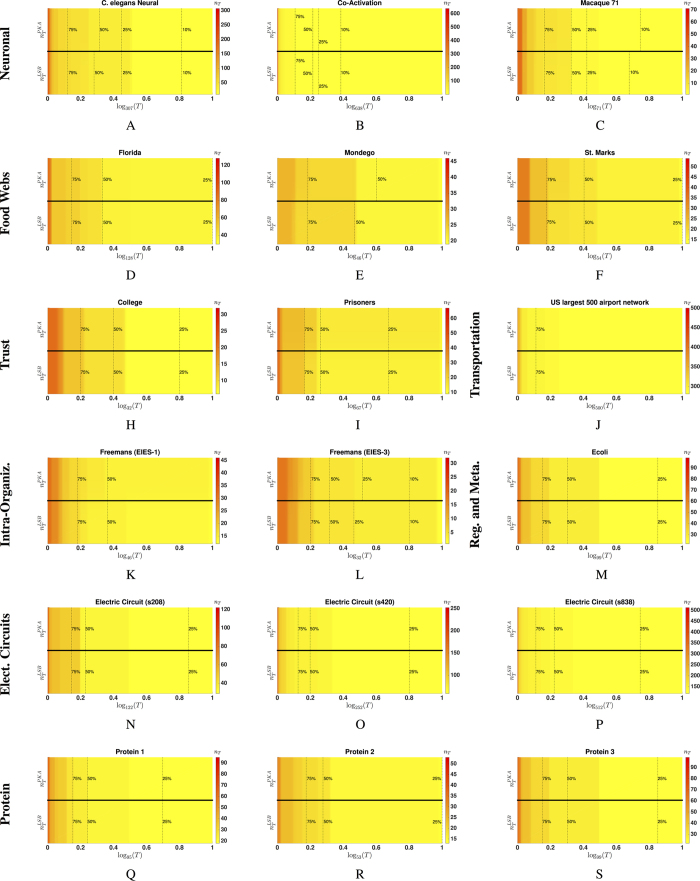
Artificial networks and their actuation spectra.

**Figure 4:**
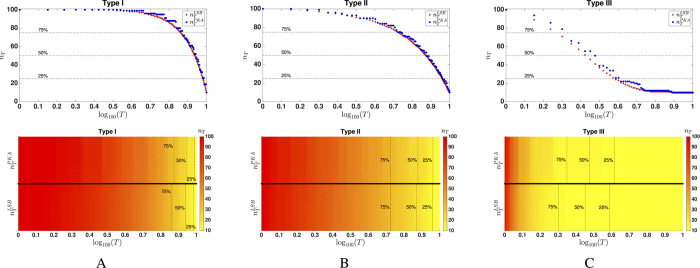
Actuation spectra of a collection of real networks.

To understand the trade-offs between the number of driving/driven nodes and the minimum time required to achieve an arbitrary network state, we introduce the notion of *actuation spectrum* of a network. Given the topology of a network, described by the structural matrix 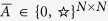, the actuation spectrum is defined by the sequence of integers 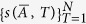, where 
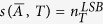
, with 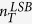 being the minimum number of driving nodes required to actuate the network such that the resulting structural controllability index is *T*, and the superscript label stands for the first letter of the authors last name in ref. 9. Alternatively, the actuation spectrum can also be defined with 
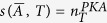
, with 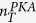 being the minimum number of driven nodes such that the resulting structural controllability index is *T*, and the superscript label stands for the first letter of the authors last name in ref. 13. Notice that for each value of the structural controllability index *T*, we have that 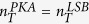 for undirected graphs, and 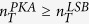 for directed graphs^13^. In Fig. 2, we depict the actuation spectrum using a heat-map where yellow (respectively, red) corresponds to a low (respectively, high) number driving nodes^9^ (denoted by 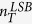) or driven nodes^13^ (denoted by 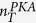) required to ensure a structural controllability index equal to *T* (in the *x*-axis). As we see in Figs 3 and 4, for most real and synthetic networks, the sequence 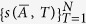 decays very fast as *T* increases (i.e., the number of driving/driven nodes required to steer the network decreases rapidly as a function of the time-to-control). Therefore, it is convenient to represent the actuation spectra using a logarithmic scale over *T*. For this purpose, we consider a logarithmic base equal to the size *N* of the network, i.e., we use log_*N*_(*T*) in *x*-axis in the actuation spectra. As a consequence, the abscissas of the actuation spectra ranges from 0 to 1, independently of the size of the network. Notice that the highest number of driving/driven nodes (darkest red in Fig. 2) is required when the structural controllability index equals 1 (i.e., 0 in the log_*N*_-scale), i.e., we steer the whole network in a single time step. In this case, it is easy to see that every single state in the network must be actuated by an input (i.e., 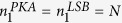, or, equivalently, 1 in the log_*N*_-scale). Similarly, the lowest number of driving/driven nodes (brightest yellow in Fig. 2) is required when we neglect time constraints, i.e., we consider the ‘standard’ minimum structural controllability problem^9^.

The representation of the actuation spectrum as a heat-map enables a visual interpretation and diagnosis of the actuation trade-offs between the number of driving/driven nodes and the structural controllability index. We illustrate this point by considering the actuation spectra of three different networks with 100 nodes, depicted in Fig. 2. First, notice that these three artificial networks require 100 driving/driven nodes (depicted by ‘dark’ red levels in the spectra) to ensure the structural controllability index to be *T* = 1 (i.e., 0 in the log_100_-scale used in the *x*-axis). In addition, the minimum number of driving/driven nodes to ensure structural controllability (without any time constraints) is equal to 10 (i.e., 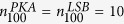 when *T* = 100, or, equivalently, 1 in the log_100_-scale used in the *x*-axis) depicted by ‘light’ yellow levels in the spectra. In Fig. 2A, we show an example of a network in which the number of driving/driven nodes decreases slowly for low values of the structural controllability index. More specifically, if we steer the network using 75 driving nodes (i.e., corresponding to 75% of the nodes of the network), we would need to actuate the network during at most 53 time steps, since the corresponding controllability index is 53 (i.e., 0.862 in log_100_-scale). In Fig. 2B, we plot the actuation spectrum of a network with a linear trade-off between the number of driving/driven nodes and the structural controllability index. In other words, if we control the network using 25 nodes, then it can be steered to any arbitrary state within 75 time steps (i.e., 0.938 in log_100_-scale). Similarly, if we control 75 nodes, then we can drive the system to any configuration within 25 time steps. Finally, in Fig. 2C, we consider a network that can be steered to any desired state in a small time window using a relatively small percentage of driving/driven nodes. More specifically, by controlling 25% of the nodes, it is possible to steer the network in at most 15 time steps (i.e., 0.588 in log_100_-scale). In addition, we observe a flat yellow region in the actuation spectrum of the network in Fig. 2C in the range 30 < *T* < 100 (i.e., 0.739–1.000 in log_100_-scale). In this flat region, there is no trade-off between the minimum number of driving/driven nodes and the structural controllability index, since 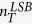 and 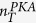 cannot be sensibly reduced by increasing the allowed time-to-control *T*.

Based on the above observations, we can readily classify networks according to their ‘agility’ using the actuation spectra. For instance, consider the following three examples: (*i*) a network with a large red region in its actuation spectrum (such as Fig. 2A) requires a large number of driving/driven nodes to steer the network to a desired state in a short time window; (*ii*) networks with an actuation spectrum (Fig. 2B) that requires a number of driving/driven control nodes that decrease affinely with the structural controllability index *T*; and (*iii*) networks with a large yellow region in their spectrum (Fig. 2C) require a small number of driving/driven nodes to steer the network within a relatively small time window. In conclusion, the faster the decrease of 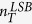 (or 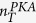) with respect to *T*, the more ‘agile’ the network is. In other words, the presence of a large yellow region in the controllability spectrum is an indication of a network being agile from a control point of view. In Figs 3 and 4, we include a variety of actuation spectra for a collection of both real and synthetic networks. In what follows, we describe a few challenges regarding the computation of the actuation spectra.

It can be formally shown that the problem of determining the minimum number of driving/driven nodes to achieve a given structural controllability index is computationally hard (see *SI Text*, section II, Theorem 4). As illustrated by Fig. 1A–C, there can potentially exist several possible combinations of disjoint unions of input cacti spanning the system digraph. Remember that the structural controllability index *T* is dominated by the cactus with the largest number of state nodes. Therefore, in order to find the minimum number of driving/driven nodes to obtain a structural controllability index *T*, we would need to consider all possible disjoint unions of spanning cacti and find the spanning cacti in which the largest cactus (in the number of state nodes) is minimized. Since this is a hard combinatorial problem, we propose a two-step approach (illustrated in Fig. 1D–F) that allows us to obtain sub-optimal results with optimality guarantees. In the first step of this approach, we search for a partition of the state digraph into a disjoint collection of subgraphs with at most *T* state vertices per subgraph, such that each subgraph in this partition is spanned by input cacti having at most *T* state vertices per cactus (see Fig. 1E for a partition of the state digraph in Fig. 1D for *T* = 8). In the second step, we determine the minimum number of driving/driven nodes required for each subgraph to ensure structural controllability, which can be achieved by solving a maximum matching problem^9^,^13^ (see Fig. 1F for the set of input nodes required for each subgraph). As a result of these two steps, we find a collection of disjoint input cacti spanning the system digraph, where each cactus contains at most *T* state vertices. Hence, if we drive the system with the union of all the driving/driven nodes corresponding to each disjoint subgraph, the network attains a structural controllability index equal to *T*. It is worth remarking that finding the partitions of a graph in the first step is a computationally challenging problem^25^. Notwithstanding, due to the wide range of practical application in which this partition problem is required, efficient algorithms are currently available to find approximate solutions incurring (consistently) in a 1–3% error^25^.

### Actuation Spectra of Artificial Complex Networks

We now examine the actuation spectra of several artificial networks, such as scale-free (SF), Erdös-Rényi (ER) and small-world (SW) networks. In Fig. 3, we include the actuation spectra of these networks for a variety of parameters and network sizes. In our illustration, we consider 200 random realizations for each one of these synthetic graphs when the number of nodes are 2500, 5000, and 10000. Figure 3A–C shows box plots and heat maps of the actuation spectra of ER graphs with average degrees 〈*k*〉 equal to 4, 6, 8, 10, and 12. In our simulations, we observe two distinct phases in these actuation spectra. One phase of the spectrum, corresponding to *T* < 5, is characterized by an abrupt decline in the required number of driving/driven nodes. In contrast, we also observe a second phase (for *T* > 5) characterized by a more gradual decrease in the number of required control nodes as *T* increases.

Remarkably, we observe that all ER networks under study present these two phases with the same boundary at *T* ≈ 5, independently of the average degree and the size of the network. This behavior can be justified based on the following fact: the minimum number 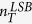 of driving nodes required to ensure a structural controllability index equal to *T* satisfies 
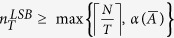
, where 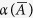 is the number of state vertices that do not belong to matching edges in the maximum matching problem (see *SI Text*, section II, Theorem 3). Notice that 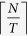 is the minimum number of subgraphs with at most *T* state vertices in a partition of the state digraph, whereas 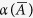 is the minimum number of driving nodes to ensure structural controllability. A possible justification for the presence of two phases in the actuation spectra of ER graphs is that, for *T* < 5, the number of partitions 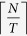 required to ensure a specific structural controllability index dominates over 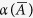. On the contrary, for *T* > 5, 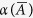 dominates over 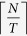, resulting in a more gradual decrease in the number of driving/driven nodes. Furthermore, we also observe that the number of driving/driven nodes increases as we decrease the average degree of the ER graph. A possible justification for this phenomenon is based on the fact that the resulting number of driving nodes counts the minimum number of paths in a decomposition of the state digraph into paths and cycles, among all possible such decompositions^9^,^13^. In particular, we observe that, as we decrease the average degree of the random graph, the minimum number of paths in the aforementioned decomposition increases.

In Fig. 3D–F, we plot the actuation spectra of scale free networks for different sizes and parameters. These actuation spectra also present two phases with boundaries at *T* ≈ 5 (the same location observed in the ER model). Furthermore, the location of this boundary is independent of the size of the network *N* and the minimum node degree *d* of the SF model. In general, we observe that in the first phase (i.e., *T* < 5), the dependency of the required number of driving/driven nodes is very weak with respect to the parameters of the synthetic network, for both the ER and the SF models. This indicates that, for low values of the structural controllability index *T*, the agility of the network does not depend strongly on the network parameters. In contrast, in the second phase (i.e., *T* > 5), we observe a stronger dependency on the network parameters. In other words, the agility of the network is more heavily influenced by the minimum node degree for large values of the structural controllability index *T*. This is consistent with previous studies, where this phenomenon was observed in the absence of time constraints in the control^9^. We also notice that the required number of driving/driven nodes decreases slower in the SF network than in the ER model (with the same average degree) as the controllability index increases. Therefore, in the second phase, SF networks are less ‘agile’ than ER graphs from a control point of view, since they can be controlled with less driving/driven nodes within the same time window.

In Fig. 3G–I, we plot the actuation spectra of SW networks for different values of the average degree *d* and the rewiring probability *p*. From our simulations, we conclude that the average degree and the rewiring probability have very little impact on the minimum number of driving/driven nodes. In contrast with the ER and SF models, the actuation spectra of the SW model present a single phase in which the number of partitions 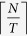 required to ensure a specific structural controllability index *T* dominates over 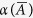. In other words, the actuation spectra decays as 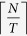. Remarkably, this decay rate is not substantially influenced by the rewiring probability (for relatively small values of *p*). In conclusion, SW networks present the fastest decrease in the number of required driving/driven nodes as the structural controllability index *T* increases. Hence, they are the most ‘agile’ among the three synthetic models under consideration, i.e., they can be steered with less driving/driven nodes within the same time window.

### Actuation Spectrum of Real Complex Networks

Apart from synthetic network models, we also study the actuation spectra of a collection of real-world networks. In Table 1, we summarize some of the main characteristics of these networks, including relevant controllability features. In Fig. 4, we include heat maps for the actuation spectra of these networks that are remarkably different from those of synthetic networks. The first row contains the spectra of several neural networks. We observe that the actuation spectrum of the C. elegans’ neural network (depicted in Fig. 4A) presents a fast decrease in the range *T* = 1 to 153 (approximately half of the network size), followed by a more gradual decrease from *T* = 154 until *T* = 300. We observe a similar behavior in the Macaque’s brain connectivity network presented in Fig. 4C, in which each node corresponds to a brain region and each edge represents white matter fiber tracts connecting pairs of regions. We notice that, even though brain connectivity networks exhibit structural characteristics similar to SW networks^26^, their corresponding actuation spectra are drastically different. In particular, we need 13% of the nodes (respectively, 3% of the nodes) to achieve a controllability index of 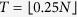 (respectively, 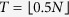) for the Macaque network, while these values are 0.3% (respectively, 0.12%) for the SW network. This observation justifies the need for better synthetic models capable of capturing controllability properties of the network, beyond simple structural features. As part of our experiments, we also analyze the actuation spectrum of the human co-activation network, where nodes represent brain regions and edges represent pairs of regions with a high level of brain activity correlation. The corresponding actuation spectrum presents a sharp gradient for low values of *T*, indicating that the human co-activation network is very ‘agile’ from a controllability point of view. In addition, we include a variety of real-world actuation spectra in Fig. 4, which are substantially different from those of the synthetic models as well.

## Discussion

In general, not only are we interested in steering a complex network towards a desired state, but also in doing so within a given time window. In this context, it is fundamental to understand the trade-offs between the number of driving/driven nodes and the time required to reach a desired state. Towards this goal, we have introduced the notion of *actuation spectrum*, which provides new insights into our ability to steer the dynamics of complex networks by taking into account the time-to-control. Nonetheless, computing the actuation spectrum of a complex network is computationally challenging; therefore, we have proposed an efficient algorithm to approximate it, while providing performance guarantees.

We have empirically analyzed the actuation spectrum of a wide variety of real and synthetic complex networks, and have found that in many cases only a small fraction of driving/driven nodes is required to steer the network to a desired state within a relatively small time window. Our numerical experiments have also unveiled the presence of a controllability phase transition in Erdös-Rényi and Scale-Free networks. In particular, the controllability properties of both networks change drastically when the structural controllability index crosses the value *T* = 5. Even though phase transitions of topological graph properties (e.g., distribution of connected components) have been widely studied, phase transitions of controllability properties are yet to be understood. Our empirical studies also reveal that, even though synthetic models are designed to present topological properties similar to those observed in real networks, their controllability properties (e.g., their actuation spectra) can be drastically different. For example, even though small-world networks have been used as models of brain networks, their actuation spectra are rather dissimilar. Despite the wide variety of synthetic network models in the literature, there is a need for new models able to replicate not only structural metrics, but also controllability properties observed in real-world networks.

## Methods

### Structural Controllability Index

In order to compute the actuation spectrum, we need to repeatedly solve the problem of finding the minimum number of driving/driven nodes given a bound on the time-to-control. Since this problem is computationally challenging (see *SI Text*, section II, Theorem 4), we propose a two-step approximation algorithm with quality guarantees. The two steps in this algorithm are the following: first, given a prescribed controllability index *T*, we partition the state digraph 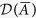 into a collection of disjoint of weakly connected subgraphs of size at most *T*. In the second step, for each subgraph, we compute the minimum number of driving/driven nodes. As a result, the total number of driving/driven nodes required to drive the network towards an arbitrary state within *T* time steps is equal to the sum of the driving/driven nodes over all subgraphs.

### Minimum Number of Driving/Driven Nodes

To compute the minimum number of driving nodes, we find a maximum matching on the bipartite graph representation of a state digraph associated with the structural matrix 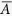. The number of driving nodes is then equal to 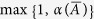, where 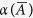 is the number of unmatched state vertices in the maximum matching^9^. To obtain the minimum number of driven nodes, we find a minimum weight maximum matching (i.e., a maximum matching with the minimum weight sum) of an augmented bipartite graph representation of the state digraph^13^, as described below. Briefly, the augmented bipartite graph consists of the bipartite graph representation of the state digraph and a collection of additional ‘slack’ nodes. In particular, we include as many slack nodes as the number of root strongly connected components of the state digraph, i.e., strongly connected components (SCCs) without incoming edges coming into them. Then, each slack node is connected to all the state nodes in one and only one root-SCC. Furthermore, a weight equal to 1 is assigned to those edges connecting state variables, and a weight equal to 2 is assigned to the edges incident to slack nodes. By finding a minimum weight maximum matching in this augmented bipartite graph, we obtain the maximum number of unmatched state vertices distributed across different root-SCCs^13^; hence, minimizing the required conditions to have a minimum number of driven nodes. Subsequently, the total number of driven nodes equals the number of unmatched vertices in the minimum weight maximum matching plus the total number of root-SCCs without an unmatched state vertex belonging to it^13^.

### Graph Partition Problem

The graph partition (GP) problem consists in determining the minimum number *κ* of weakly connected subgraphs of 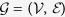, where the set of subgraphs 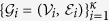 satisfy the following conditions: (*i*) 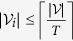, (*ii*) 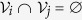 for *i* ≠ *j*, and (*iii*) 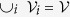. Even though the GP problem is known to be NP-hard, it is possible to efficiently approximate the solution to this problem using polynomial-time algorithms^25^. One of the most successful tools to approximate the GP problem is implemented in a publicly available software package is called METIS, and it is used by us to obtain the actuation spectra. In practice, METIS has consistently shown to lead to only a 1–3% of partitions that do not satisfy 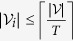.

## Additional Information

**How to cite this article**: Pequito, S. *et al*. Trade-offs between driving nodes and time-to-control in complex networks. *Sci. Rep.* **7**, 39978; doi: 10.1038/srep39978 (2017).

**Publisher's note:** Springer Nature remains neutral with regard to jurisdictional claims in published maps and institutional affiliations.

## Supplementary Material

Supplementary Information

## Figures and Tables

**Table 1:** Properties of real networks analyzed in this paper.

Label	Name	*N*	*E*	〈*k*〉	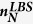	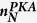	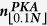	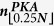	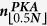	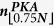
Neuronal										
1	C. Elegans	307	2657	8.6547	10	10	55	43	29	11
2	Co-Activation	638	37250	58.3856	1	1	11	5	3	2
3	Macaque 71	71	746	10.5070	1	1	15	9	3	2
Food Webs										
4	Florida	128	2106	16.4531	30	30	51	42	35	34
5	Mondego	46	400	8.6957	19	19	27	23	20	20
6	St. Marks	54	356	6.5926	13	13	23	19	15	15
Trust										
7	College	32	96	3.0000	6	6	17	9	6	6
8	Prisioners	67	182	2.7164	9	11	24	20	14	14
Transportation										
9	US largest 500 airport	500	2980	5.9600	281	281	291	286	282	281
Intra-Orgazinational										
10	Freemans (EIES-1)	46	695	15.1087	12	13	23	16	14	14
11	Freemans (EIES-3)	32	460	14.3750	1	1	14	4	3	3
Reg. and Metabolic										
12	Ecoli	99	212	2.1856	22	22	39	28	27	22
Electric Circuit										
13	s208	122	189	1.5492	29	29	47	33	30	30
14	s420	252	399	1.5833	59	59	73	66	62	62
15	s838	512	819	1.5996	119	119	140	128	125	123
Protein										
16	Protein 1	95	213	2.2421	18	18	33	24	21	19
17	Protein 2	53	123	2.3208	13	13	23	18	14	14
18	Protein 3	99	212	2.2316	22	22	39	28	27	22

Legend: *n* denotes the number of nodes, *E* denotes the number of directed edges, 〈*k*〉 denotes the average degree, 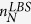 the number of driving nodes and 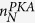 the number of driven nodes to ensure structural controllability, and 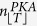 the number of driven nodes required if the structural controllability index is set to be equal to 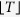, with *T* = 0.1*N*, 0.25*N*, 0.5*N*, 0.75*N*.
