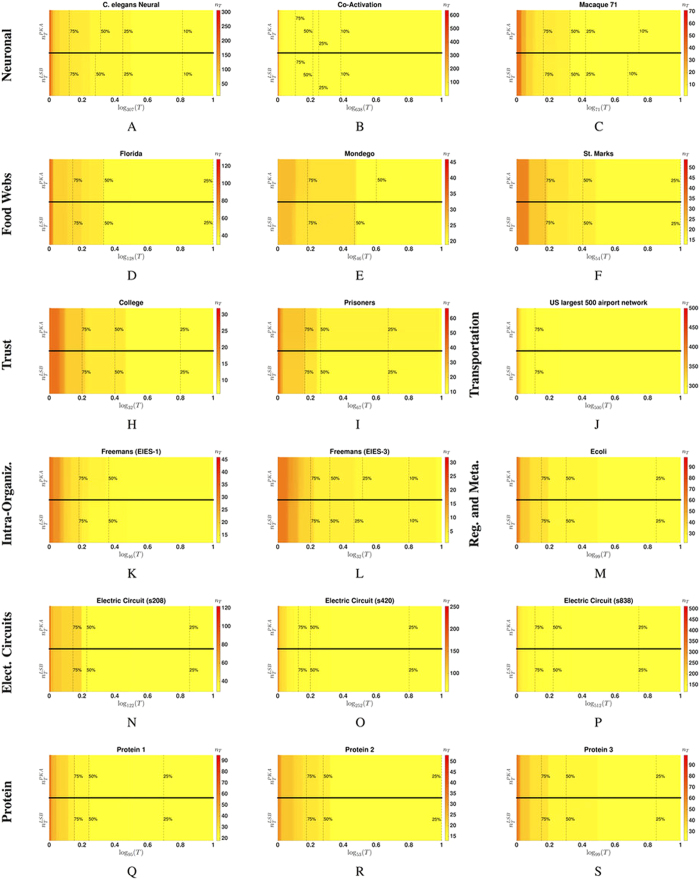# Corrigendum: Trade-offs between driving nodes and time-to-control in complex networks

**DOI:** 10.1038/srep43194

**Published:** 2017-03-09

**Authors:** Sérgio Pequito, Victor M. Preciado, Albert-László Barabási, George J. Pappas

Scientific Reports 7: Article number: 3997810.1038/srep39978; published online: 01 05 2017; updated: 03 09 2017

This Article contains an error in the order of the Figures. Figures 1, 2, 3 and 4 were published as Figures 2, 4, 1 and 3 respectively. The correct Figures appear below. The Figure legends are correct.

## Figures and Tables

**Figure 1:**
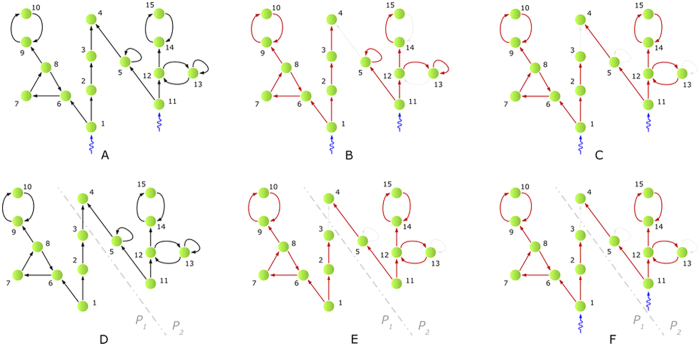


**Figure 2:**
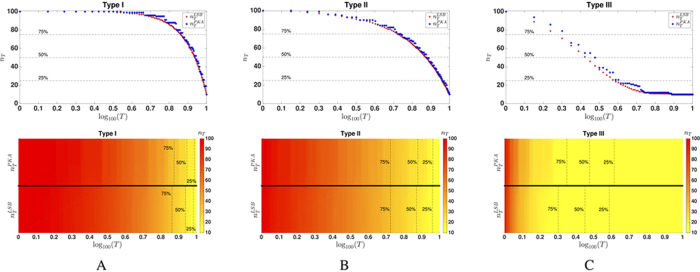


**Figure 3:**
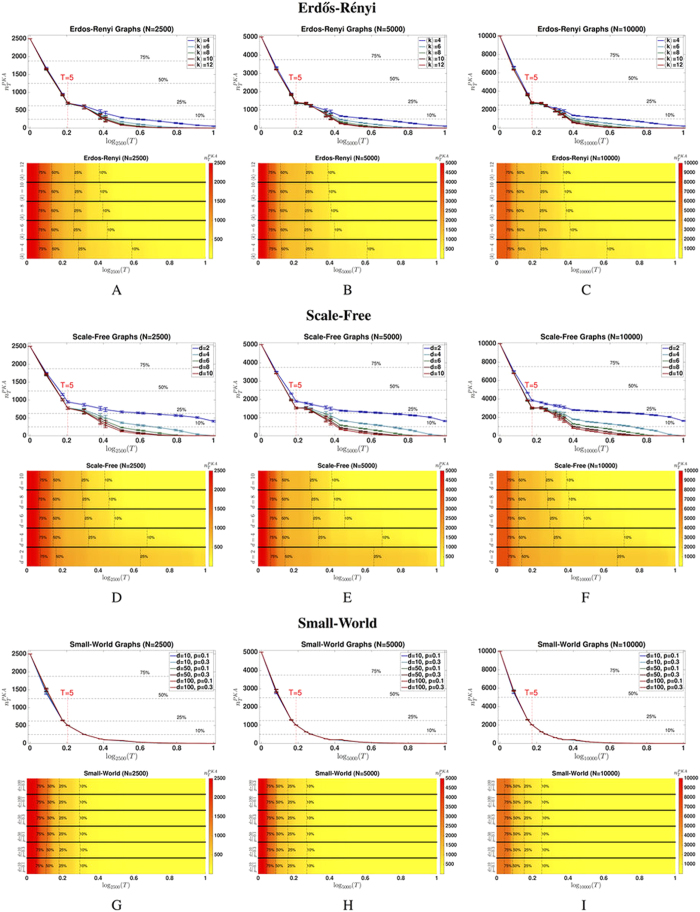


**Figure 4:**