# Supplementary material for: Trade-offs between driving nodes and time-to-control in complex networks
Source: Sci Rep. 2017 Jan 5;7:39978. doi: 10.1038/srep39978 (PMC5215470; doi:10.1038/srep39978)
Supplement: Supplementary Information [file srep39978-s1.pdf]

Trade-offs between driving nodes and time-to-control in complex networks – Supplementary Information –

Sérgio Pequito † Victor M. Preciado † Albert-Lászl Barabási ‡ George J. Pappas †

CONTENTS

I	PRELIMINARIES AND TERMINOLOGY	1
II	MAIN RESULTS	5
References		11

I. PRELIMINARIES AND TERMINOLOGY

In this section, we first review some concepts from control theory [1], graph theory, and structural systems theory [2], [3]. We also include some notions of computational complexity needed in our analysis [4].

Controllability Index

Consider a dynamical network modeled as the following linear discrete time-invariant system:

$$x[t+1] = Ax[t] + Bu[t], \; t = 0, 1, \ldots, \tag{1}$$

where $x[t] \in \mathbb{R}^N$ is a vector containing the states of all the nodes in the network at time t, $x[0] = x_0$ is the initial state, and $u[t] \in \mathbb{R}^P$ is the value of the P-dimensional input signal injected in the network at time t. The matrix $A \in \mathbb{R}^{N\times N}$ is the state matrix, which captures the dynamic interdependencies among nodes; the matrix $B \in \mathbb{R}^{N\times P}$ is the input matrix, which identifies those nodes that are actuated by an

†Department of Electrical and Systems Engineering, School of Engineering and Applied Science, University of Pennsylvania, Philadelphia, Pennsylvania 19104, USA.

‡ Center for Complex Network Research and Departments of Physics, Computer Science and Biology, Northeastern University, Boston, Massachusetts 02115, USA; Center for Cancer Systems Biology, Dana-Farber Cancer Institute, Boston, Massachusetts 02115, USA; and Department of Medicine, Brighamand Women's Hospital, Harvard Medical School, Boston, Massachusetts 02115, USA.

external input signal. In addition, let us assume that the input matrix $B \in \mathbb{R}^{N \times P}$ has rank P (i.e., full column rank). Notice that if B does not have full column rank, then some columns of B can be written as a linear combination of the remaining ones. Consequently, removing linearly dependent columns of B (and their corresponding inputs) would not affect our ability to control the network. If the system in (1) is controllable, then its controllability matrix

$$\mathcal{C}(A, B; N) = [B \ AB \ \cdots \ A^{N-1}B]$$

has rank N (i.e., N linearly independent columns). In what follows, we introduce the concept of controllability index to account for the time-to-control.

Let b_i be the ith column of B, then the controllability matrix $\mathcal{C}(A, B; N)$ can be written as follows:

$$\mathcal{C}(A, B; N) = [b_1 \ \cdots \ b_P \mid Ab_1 \ \cdots \ Ab_P \mid \ \cdots \ \mid A^{N-1}b_1 \ \cdots \ A^{N-1}b_P].$$

To define the controllability index, for each $i \in \{1, \ldots, P\}$, we define the set of column vectors $\mathcal{S}_i = \{A^{j-1}b_i\}_{j=1}^{\tau_i}$ where τ_i is the maximum integer for which the set of column vectors in $\mathcal{S}_i$ is linearly independent. Therefore, it can be proved that if $\mathcal{C}(A, B; N)$ has rank N, then

$$\tau_1 + \tau_2 + \ldots + \tau_P = N.$$

The *controllability index* of the pair (A, B), describing the dynamical network in (1), is defined as [1]

$$\tau(A, B) = \max\{\tau_1, \tau_2, \ldots, \tau_P\}.$$

Equivalently, if (A, B) is controllable, the controllability index $\tau(A, B)$ is the least integer T such that

$$\text{rank}(\mathcal{C}(A, B; T)) = N.$$

From a control point of view, the controllability index $\tau(A, B)$ is equal to the minimum number of time steps required to steer the system from an initial state x_0 to an arbitrary desired state $x_d \in \mathbb{R}^N$. In particular, if the system is controllable in T time steps, and the initial state is the origin (i.e., $x_0 = 0$), then the input signal $\{u[t]\}_{t=0}^{T-1}$ that steers the system to x_d can be explicitly computed as [5]

$$u_{0:T-1} = \mathcal{C}(A, B; T)^{\intercal} \left[\mathcal{C}(A, B; T)\, \mathcal{C}(A, B; T)^{\intercal}\right]^{-1} x_d, \tag{2}$$

where $u_{0:T-1}^{\intercal} = [u[0]^{\intercal}, u[1]^{\intercal}, \ldots, u[T-1]^{\intercal}]$ is a vector in $\mathbb{R}^{TP}$ containing a concatenation of the input signal. Notice that, for $T \geq \tau(A, B)$, the matrix inside the brackets in (2) is invertible and $u_{0:T-1}$ is well-defined.

Graph Theory and Structural Systems

The following standard terminology and notions from graph theory can be found, for instance, in [3]. Let $\mathcal{D}(\bar{A}) = (\mathcal{X}, \mathcal{E}_{\mathcal{X},\mathcal{X}})$ be the *state digraph* corresponding to the digraph representation of $\bar{A} \in \{0, \star\}^{N\times N}$ (i.e., the structural matrix associated with A in (1)), where the node set $\mathcal{X}$ has its nodes labeled by the state variables (also referred to as *state nodes*) and $\mathcal{E}_{\mathcal{X},\mathcal{X}} = \{(x_i, x_j) \colon A_{ji} \neq 0\}$ denotes the set of edges connecting state nodes. Similarly, we define the *system digraph* $\mathcal{D}(\bar{A}, \bar{B}) = (\mathcal{X} \cup \mathcal{U}, \mathcal{E}_{\mathcal{X},\mathcal{X}} \cup \mathcal{E}_{\mathcal{U},\mathcal{X}})$, where $\bar{B} \in \{0, \star\}^{N\times P}$ represents the structural matrix associated with B in (1), $\mathcal{U}$ represents the set of P nodes labeled by the input variables (also referred to as *input nodes*), and $\mathcal{E}_{\mathcal{U},\mathcal{X}} = \{(u_i, x_j) \colon \bar{B}_{ji} \neq 0\}$.

A digraph $\mathcal{D}_s = (\mathcal{V}_s, \mathcal{E}_s)$ with $\mathcal{V}_s \subset \mathcal{V}$ and $\mathcal{E}_s \subset (\mathcal{V}_s \times \mathcal{V}_s) \cap \mathcal{E}$ is called a *subgraph* of $\mathcal{D} = (\mathcal{V}, \mathcal{E})$. If $\mathcal{V}_s = \mathcal{V}$, then $\mathcal{D}_s$ is said to *span* $\mathcal{D}$. Two digraphs are *disjoint* if they do not share any node. A subgraph of $\mathcal{D}_s$ with some property P (e.g., being connected) is *maximal* if there is no other subgraph containing $\mathcal{D}_s$ satisfying property P. A sequence of directed edges $\{(v_1, v_2), (v_2, v_3), \cdots, (v_{l-1}, v_l)\}$, in which the nodes $v_1, \ldots, v_{l-1}$ are all distinct, is called *an elementary path* from v_1 to v_l, and v_1 (respectively, v_l) is called the *root* (respectively, the *end*) of the path. An elementary path is said to be *open* if $v_1 \neq v_l$. An elementary path for which $v_1 = v_l$ is called a *cycle* (in particular, a node with an edge to itself, i.e., a *self-loop*, is a cycle).

In addition, a digraph $\mathcal{D}$ is said to be strongly connected if there exists an elementary path between any pair of nodes. A *strongly connected component* (SCC) is a maximal subgraph $\mathcal{D}_S = (\mathcal{V}_S, \mathcal{E}_S)$ of $\mathcal{D}$ for which the following property is satisfied: for every pair of nodes $v, w \in \mathcal{V}_S$, there exists a path in $\mathcal{D}_S$ from v to w. Similarly, if the graph is undirected, a strongly connected graph is simply said to be *connected*. A digraph is said to be *weakly connected* if, after disregarding edge directions, the resulting graph is connected. A *directed tree* is a digraph if, after disregarding edge directions, the resulting graph is connected and does not contain cycles. Furthermore, a collection of disjoint directed trees is referred to as a *directed forest*. Finally, the graph partition (GP) problem consists in determining κ weakly connected subgraphs of $\mathcal{G} = (\mathcal{V}, \mathcal{E})$, where the set of subgraphs $\{\mathcal{G}_i = (\mathcal{V}_i, \mathcal{E}_i)\}_{i=1}^{\kappa}$ satisfy the following conditions: (*i*) $|\mathcal{V}_i| \leq \left\lceil \frac{|\mathcal{V}|}{\kappa} \right\rceil$, (*ii*) $\mathcal{V}_i \cap \mathcal{V}_j = \emptyset$ for $i \neq j$, and (*iii*) $\bigcup_i \mathcal{V}_i = \mathcal{V}$.

In what follows, we define specific subgraphs that are relevant in structural system theory [2], [3]. Given a state digraph $\mathcal{D}(\bar{A})$ and a system digraph $\mathcal{D}(\bar{A}, \bar{B})$, we define the following special subgraphs [6]:

- *State Stem* - An isolated node or an open elementary path, composed exclusively of state nodes.
- *Input Stem* - An input node linked to the root of a state stem.
- *State Cactus* - Defined recursively as follows: A state stem is a state cactus. A state cactus connected by an edge to a (disjoint) cycle is also a state cactus.
- *Input Cactus* - Defined recursively as follows: An input stem with at least one state node is an input

cactus. An input cactus connected by an edge to a disjoint cycle of state nodes is also an input cactus. ∘

The root and the end of a stem are also called the root and the end of the associated cactus, respectively. Note that, by definition, an input cactus may have an input node linked to several state nodes. In particular, an input node may connect to the root of a state stem and to one or more states in a cycle.

A structural system defined by the pair of structural matrices $(\bar{A}, \bar{B})$ is said to be *structurally controllable* if there exists a pair (A_0, B_0) of real matrices with the same structure as $(\bar{A}, \bar{B})$ such that (A_0, B_0) is controllable [2]. In particular, it can be shown that if such pair (A_0, B_0) exists then almost all possible pairs (A', B') with the same structure as $(\bar{A}, \bar{B})$ are controllable [7]. The structural controllability of a system may be characterized as follows:

Theorem 1 ([2]): Consider the structural matrices $\bar{A} \in \{0, \star\}^{N\times N}$ and $\bar{B} \in \{0, \star\}^{N\times P}$ associated with the system described by (1). The following statements are equivalent:

(*i*) the structural system $(\bar{A}, \bar{B})$ is structurally controllable;

(*ii*) the digraph $\mathcal{D}(\bar{A}, \bar{B})$ is spanned by a disjoint union of input cacti. ⋄

Finally, the input vertices in the system digraph required to ensure structural controllability, also referred to as *driving nodes*, can be characterized in terms of *state unmatched nodes* associated with a maximum matching of the bipartite representation of the state digraph (see [8] for details). In particular, the minimum number of driving nodes (or, equivalently, the minimum number of state unmatched nodes) is related with the so called *term-rank* of $\bar{A} \in \{0, \star\}^{N\times N}$, denoted by $\sigma(\bar{A})$ and defined as the maximum number of non-zero diagonal elements in any of the matrices resulting from a permutation of rows and columns of $\bar{A}$. Therefore, the minimum number of driving nodes can be described as

$$\alpha(\bar{A}) = N - \sigma(\bar{A}). \tag{3}$$

Computational Complexity

In what follows, we introduce some concepts of computational complexity theory [9]. This theory allows the classification of (computational) problems into complexity classes. In particular, we can classify *decision problems*, i.e., problems with a "yes" or "no" answer. Furthermore, if there exists a procedure/algorithm that obtains the correct answer of a decision problem in a number of steps that is bounded by a polynomial in the size of the input data, then the algorithm is referred to as an *efficient* or *polynomial-time* solution, and the decision problem is said to be polynomially solvable. A decision problem is said to be in NP (i.e., nondeterministic polynomial) if any possible solution instance can be verified using a polynomial procedure. It is easy to see that any problem that is polynomially solvable is also in NP, although, there are some problems in NP for which it is unclear whether polynomial solutions exist or not. These latter problems are referred to as being NP-complete. Consequently, the class of NP-complete problems is

the *hardest* among the NP problems, i.e., those that are verifiable using polynomial algorithms, but no polynomial algorithms to solve them are known to exist. Although the above classification is intended for decision problems, it can be immediately extended to general optimization problems by noticing that every optimization problem can be posed as a decision problem. More precisely, given a minimization problem, we can pose the following decision problem: Is there a solution to the minimization problem that is less than or equal to a prescribed value? On the other hand, if the solution to the optimization problem is known, then its decision version can be trivially addressed. Consequently, if a (decision) problem is NP-complete, then the associated optimization problem is referred to as being NP-hard. We suggest the reader to [4] for an introduction to the topic of computational complexity.

In the next section, we show that the problem of finding the minimum number of driven nodes to ensure a structural controllability index equal to T is NP-hard. The NP-hardness is demonstrated by polynomially reducing this problem to a well known NP-hard problem, in particular, the GP problem introduced above. Even though polynomial complexity algorithms able to solve general instances of the GP problem are unlikely to exist, it is possible to approximate its solution using polynomial-time algorithms (with some optimality guarantees). One of the most successful software tools to approximate the GP problem is called METIS [10], described in further detail in the next section.

II. MAIN RESULTS

In this section, we formally introduce the structural counterpart of the controllability index, the *structural controllability index*. In Theorem 2, we provide a graph-theoretical characterization of the structural controllability index. In Theorem 3, we provide a lower bound on the structural controllability index in terms of the state unmatched nodes and graph partitions. In Theorem 4, we show that the problem of computing the minimum number of driven nodes under constraints in the time-to-control is NP-hard. Subsequently, we propose an efficient approximation approach, described in Algorithm 1. This algorithm provides us with an upper bound on the structural controllability index. Comparing this upper bound with the lower bound in Theorem 3, we can assess the quality of our approximation. Furthermore, the proposed algorithm leverages existing tools that consistently achieve approximate solutions with optimality guarantees.

This structural controllability index is defined as follows [11], [12]: Consider the structural matrices $\bar{A} \in \{0, \star\}^{N\times N}$ and $\bar{B} \in \{0, \star\}^{N\times P}$, where the entries are either 0 (i.e., there is no edge between two nodes), or an unknown nonzero entry (i.e., there is an edge between two nodes with an arbitrary weight) denoted by $\star$. In other words, the matrices $\bar{A}$ and $\bar{B}$ characterize the topology of the system digraph, when the weights can take any arbitrary value. Given a structural state matrix $\bar{A}$ and a structural input matrix $\bar{B}$, we say that the corresponding structural system is structurally controllable with index T if there

exists a pair of real matrices (A, B) corresponding to a weighted realization of the system digraph such that the controllability index of (A, B) is equal to T. In other words, we can find a (weighted) network with a system digraph matching the topology described by the pair $(\bar{A}, \bar{B})$ such that it can be controlled in (at least) T time steps. This value of T is called the structural controllability index, which we denote by $\bar{\tau}(\bar{A}, \bar{B})$.

To formalize this concept, we need to introduce the notion of *generic rank* of the partial controllability matrix $\mathcal{C}(A, B; T)$ defined as follows:

$$\rho(\bar{A}, \bar{B}; T) = \max_{A' \in [\bar{A}], B' \in [\bar{B}]} \text{rank } (\mathcal{C}(A', B'; T)),$$

where $[\bar{M}] = \{M \in \mathbb{R}^{N_1 \times N_2} : M_{i,j} = 0 \text{ if } \bar{M}_{i,j} = 0\}$ for a structural matrix $\bar{M} \in \{0, \star\}^{N_1 \times N_2}$. Using similar arguments to those provided in [7], it readily follows that if there exists a pair (A_0, B_0) with controllability index T, then almost all possible pairs (A', B'), with $A' \in [\bar{A}]$ and $B' \in [\bar{B}]$, have controllability index T. Therefore, the *structural controllability index* $\bar{\tau}(\bar{A}, \bar{B})$ of the pair $(\bar{A}, \bar{B})$ is given by

$$\bar{\tau}(\bar{A}, \bar{B}) = \min\{T \in \{1, \ldots, N\} \colon \rho(\bar{A}, \bar{B}; T) = N\}. \tag{4}$$

In what follows, we define the *term rank* of a structural matrix, which is useful to provide a graph-theoretical interpretation of the structural controllability index. Consider a partial structural controllability matrix of order T, given by

$$\bar{\mathcal{C}}(\bar{A}, \bar{B}; T) = [\bar{B} \;\; \bar{A}\bar{B} \;\; \ldots \;\; \bar{A}^{T-1}\bar{B}],$$

where the entries of the product of two structural matrices $\bar{M}_1$ and $\bar{M}_2$ satisfy $[\bar{M}_1\bar{M}_2]_{i,j} = \star$, if there exists an integer k such that $[\bar{M}_1]_{i,k} = \star$ and $[\bar{M}_2]_{k,j} = \star$; and $[\bar{M}_1\bar{M}_2]_{i,j} = 0$ otherwise. Given a rectangular structural matrix $\bar{R}$, we define the operator $\overline{\text{Tr}}(\bar{R})$ as the number of entries in the main diagonal equal to $\star$. The term-rank then is defined by

$$r(\bar{A}, \bar{B}; T) = \max_{P_1, P_2} \overline{\text{Tr}}(P_1 \, \bar{\mathcal{C}}(\bar{A}, \bar{B}; T) \, P_2),$$

where P_1 and P_2 and two permutation matrices of appropriate dimensions.

Based on these concepts, the structural controllability index can be characterized as follows:

Lemma 1 ([13]): The structural controllability index of $(\bar{A}, \bar{B})$, defined in (4), is the minimum value of $T \in \{1, \ldots, N\}$ such that the following two conditions are satisfied:

(a) $r(\bar{A}, \bar{B}; T) = N$; and

(b) every state node in $\mathcal{D}(\bar{A}, \bar{B})$ is the end of a directed path that starts in some input node. $\diamond$

Therefore, from Lemma 1 it readily follows that verifying these two conditions can be done in polynomial time. More specifically, Condition (a) in Lemma 1 can be verified by resorting to a maximum

matching problem, and Condition (b) in Lemma 1 can be verified by performing a depth-first search in $\mathcal{D}(\bar{A},\bar{B})$. Consequently, the overall computational complexity is $\mathcal{O}(\max\{\sqrt{n}m, n+m\})$, where n and m are the number of nodes and edges in $\mathcal{D}(\bar{A},\bar{B})$, respectively.

Problem Statement

Let us define the $N\times N$ structural identity matrix $\bar{\mathbb{I}}_N$ entry-wise as $[\bar{\mathbb{I}}_N]_{i,i}=\star$ for all $i=1,\ldots,N$; $[\bar{\mathbb{I}}_N]_{i,j}=0$ for all $i\neq j$. Given a set $\mathcal{J}\subset\{1,\ldots,N\}$, we denote by $\bar{\mathbb{I}}_N(\mathcal{J})$ the structural matrix containing the set of columns of $\bar{\mathbb{I}}_N$ indexed by $\mathcal{J}$. Hereafter, we address the following problem:

Problem $\mathcal{P}_1$: Given $\bar{A}\in\{0,\star\}^{N\times N}$ and $T\in\{1,\ldots,N\}$, determine the set $\mathcal{J}\subset\{1,\ldots,N\}$ with minimum cardinality $|\mathcal{J}|$ such that $(\bar{A},\bar{\mathbb{I}}_N(\mathcal{J}))$ is structurally controllable with index T. ∘

The optimal set $\mathcal{J}\subset\{1,\ldots,N\}$ represents the minimal set of *dedicated* driving nodes, i.e., the set of input nodes that are connected to only one state node, which we will refer to as *driven* (state) nodes. A similar problem can be posed to determine the minimum number of driving nodes (i.e., not necessarily dedicated), which can be immediatly obtain from the solution to problem $\mathcal{P}_1$, as described in [3]. Now, we provide a novel graph-theoretic interpretation of the structural controllability index.

Theorem 2: A pair of structural matrices $(\bar{A},\bar{B})$ is structurally controllable with index T if and only if the system digraph $\mathcal{D}(\bar{A},\bar{B})$ is spanned by a disjoint union of input cacti, where every input cactus contains at most T state nodes. ◇

Proof: If $\mathcal{D}(\bar{A},\bar{B})$ is spanned by a disjoint union of p input cacti $\mathcal{C}=\{\mathcal{C}_i\}_{i=1}^{p}$, where every input cactus contains at most T state nodes, then we can remove the edges of $\mathcal{D}(\bar{A},\bar{B})$ (or equivalently, set to zero the free parameters associated with these edges) that do not belong to any of the input cacti in $\mathcal{C}$. Therefore, we obtain p disjoint sub-systems $(\bar{A}(\mathcal{C}_i),\bar{B}(\mathcal{C}_i))$, where $\bar{M}(\mathcal{C}_i)$ consists in the submatrix of $\bar{M}$ with the columns and rows associated with the nodes in $\mathcal{C}_i$. Subsequently, invoking Theorem 1, it follows that each subsystem is structurally controllable, and, in particular, $\bar{\tau}(\bar{A}(\mathcal{C}_i),\bar{B}(\mathcal{C}_i))\leq T$ for all $i=1,\ldots,p$. Hence, by definition of structural controllability index it follows that $\bar{\tau}(\bar{A},\bar{B})=T$.

If the pair $(\bar{A},\bar{B})$ is structurally controllable with index T, then it is also structurally controllable, and, in particular, it has to be spanned by a disjoint union of p input cacti $\mathcal{C}=\{\mathcal{C}_i\}_{i=1}^{p}$. Suppose, by contradiction, that there exist no decomposition where all input cacti contain at most T state nodes. Then, it follows that in any decomposition there exists a cactus $\mathcal{C}_j$ that has more than T state nodes. Consider two permutation matrices P and P' of appropriate dimensions, such that $\bar{A}'=P^{\intercal}\bar{A}P$ has diagonal blocks corresponding to $\bar{A}(\mathcal{C}_i)$ for $i=1,\ldots,p$, and $\bar{B}'=P^{\intercal}\bar{B}P'$ is such that the i-th column of $\bar{B}$ corresponds to $\bar{B}(\mathcal{C}_i)$. Furthermore, let $[\bar{B}'\ \bar{A}'\bar{B}'\ \ldots\ (\bar{A}')^{T-1}\bar{B}']_j$ be the row-block of $[\bar{B}'\ \bar{A}'\bar{B}'\ \ldots\ (\bar{A}')^{T-1}\bar{B}']$ associated with $\mathcal{C}_i$, i.e., its rows are those indexed by the rows of $(\bar{A}(\mathcal{C}_j),\bar{B}(\mathcal{C}_j))$. Therefore, it follows

that the j-th block-row of $[\bar{B}' \ \bar{A}'\bar{B}' \ \ldots \ (\bar{A}')^{T-1}\bar{B}']$ is such that no two permutation matrices P_1, and P_2 exist such that $\bar{\text{Tr}}(P_1[\bar{B}' \ \bar{A}'\bar{B}' \ \ldots \ (\bar{A}')^{T-1}\bar{B}']_j P_2)$ equals the number of rows, which implies that $r(\bar{A}', \bar{B}'; T) < N$. Consequently, we obtain that $\bar{\tau}(\bar{A}', \bar{B}') > T$, or, equivalently, $\bar{\tau}(\bar{A}, \bar{B}) > T$ (since the structural controllability index is invariant with respect to permutation operations), which leads to a contradiction, and the result follows. ■

As a corollary to Theorem 2, we can obtain the following known result:

Corollary 1 ([14]): Let $\bar{A} \in \{0, \star\}^{N\times N}$ and $\bar{B} \in \{0, \star\}^{N\times P}$, with $\bar{A}_{ii} = \star$ for all $i = 1, \ldots, N$, i.e., every state node in $\mathcal{D}(\bar{A}, \bar{B})$ has a self-loop. Also, let $\{\mathcal{F}_i\}_{i\in\mathcal{I}}$ be the collection of all forests spanning $\mathcal{D}(\bar{A}, \bar{B})$ containing only directed trees rooted in input nodes, where $\mathcal{I}$ contains the indices of such spanning forests. Further, a spanning forest $\mathcal{F}_i$ contains $p_i \in \mathbb{N}$ directed trees, whose collection we denote by $\mathcal{F}_i = \{\mathcal{T}_j^i\}_{j=1}^{p_i}$. Then, the structural controllability index can be defined as follows:

$$\bar{\tau}(\bar{A}, \bar{B}) = \min_{i\in\mathcal{I}} \max_{\mathcal{T}\in\mathcal{F}_i} \ |\mathcal{T}|_s,$$

where $|\mathcal{T}|_s$ denotes the number of state nodes in the tree $\mathcal{T}$ rooted in an input node. ◇

As a consequence of Theorem 2, a lower bound to the minimum number of driving nodes can be obtained as follows:

Theorem 3: Given the structural matrix $\bar{A} \in \{0, \star\}^{N\times N}$, the minimum number of driving nodes n_T^{LSB} required to ensure a structural controllability index equal to $T \in \{1, \ldots, N\}$ satisfies the following inequality

$$n_T^{LSB} \geq \max\left\{\left\lceil \frac{N}{T} \right\rceil, \alpha(\bar{A})\right\},$$

where $\alpha(\bar{A})$ is defined in (3). ◇

Therefore, if we want to ensure structural controllability (i.e., the structural controllability index is equal to N), then we obtain $n_N^{LSB} = \max\{1, \alpha(\bar{A})\}$ as prescribed in [8]. As already mentioned, finding the minimum number of driving/driven nodes to ensure a given controllability index is NP-hard [13], [15], [16]. In what follows, we provide an alternative proof to the former that relies on the GP, later used to obtain an approximate solution to our problem.

Theorem 4: Problem $\mathcal{P}_1$ is NP-hard. ◇

Proof: To show that $\mathcal{P}_1$ is NP-hard, we need to show that there exists a polynomial reduction from a problem that is known to be NP-hard to our problem. Towards this goal, we consider the graph partitioning (GP) problem, which is known to be NP-hard. Let $\mathcal{G} = (\mathcal{V}, \mathcal{E})$ be a connected undirected graph, then the GP problem aims to determining the minimum decomposition of $\mathcal{G}$ into p connected undirected graphs $\mathcal{G}_i = (\mathcal{V}_i, \mathcal{E}_i)$, with $i \in \{1, \ldots, p\}$, such that $|\mathcal{V}_i| \leq \left\lceil \frac{|\mathcal{V}|}{T} \right\rceil$, $\mathcal{V}_i \cap \mathcal{V}_j = \emptyset$ for $i \neq j$, and $\bigcup_{i=1}^{p} \mathcal{V}_i = \mathcal{V}$. Now,

consider $\mathcal{P}_1$, where $\bar{A} = \bar{A}(\mathcal{G})$ is described as follows:

$$[\bar{A}(\mathcal{G})]_{i,j} = \begin{cases} \star \text{ , if } (j,i) \in \mathcal{E} \text{ or } i = j, \\ 0 \text{ , otherwise.} \end{cases}$$

Therefore, $\mathcal{D}(\bar{A})$ is a strongly connected digraph where every state node has a self-loop. If we assume that $\mathcal{J}^*$ is a solution to $\mathcal{P}_1$, then, as a consequence of Corollary 1, it follows that each dedicated input is the root of a tree with at most T state nodes. Let $\{\mathcal{T}_i\}_{i=1}^{|\mathcal{J}^*|}$ denote the collection of such trees, where $\mathcal{T}_i = (\mathcal{X}_i \cup \{u_i\}, \mathcal{E}_{\mathcal{X}_i,\mathcal{X}_i} \cup \{u_i, x_i\})$, where (without loss of generality) x_i is the only state variable to which the input has a connection to. Therefore, it readily follows that $\mathcal{G}_i = (\mathcal{V}_i, \mathcal{E}_i)$, where $\mathcal{V}_i = \mathcal{X}_i$ and $\mathcal{E}_i = \mathcal{E}_{\mathcal{X}_i,\mathcal{X}_i}$, corresponds to a subgraph i in a partition of $\mathcal{G}$. In other words, by solving $\mathcal{P}_1$ with $\bar{A}$ as described above, we will be able to obtain a solution to the GP problem; hence, our problem is at least as difficult as the GP problem, i.e., problem $\mathcal{P}_1$ is NP-hard. ∎

Computational complexity theory can also help us to establish some strategies to find the solution to $\mathcal{P}_1$ by reduction to other well-known NP-hard problems, which can then be leveraged to obtain approximate solutions to Problem $\mathcal{P}_1$. This is what we will do next, by polynomially reducing $\mathcal{P}_1$ to a GP problem, under certain assumptions.

Theorem 5: Let $\bar{A}$ be symmetric structural matrix with zero-free diagonal, and $\mathcal{G}$ be the undirected graph associated with $\mathcal{D}(\bar{A})$, which we assume to be strongly connected. Let $\mathcal{J}^*$ contain the indices of exactly one node from each subgraph in a partition of $\mathcal{G}$, where each subgraph contains at most T nodes. Then $\mathcal{J}^*$ is a solution to $\mathcal{P}_1$. ◇

Proof: Consider a partition of $\mathcal{G}$ into a collection of subgraphs $\{\mathcal{G}_i = (\mathcal{X}_i, \mathcal{E}_{\mathcal{X}_i,\mathcal{X}_i})\}_{i=1}^{p}$ with at most T nodes. The proof follows by noticing that $\mathcal{D}_i = (\mathcal{X}_i \cup \{u_i\}, \mathcal{E}_{\mathcal{X}_i,\mathcal{X}_i} \cup \{u_i, x_{\alpha_i}\})$, where $x_{\alpha_i} \in \mathcal{X}_i$, is spanned by a directed tree rooted in u_i. Let $\{\mathcal{T}_i\}_{i=1}^{|\mathcal{J}^*|}$, where $\mathcal{J}^* = \cup_{i=1}^{p} \alpha_i$, represent the collection of these directed trees; then, the first condition in Lemma 1 yields. In addition, the second condition of the same theorem holds by assumption, since $\mathcal{D}(\bar{A})$ is spanned by cycles, due to the self-loops corresponding to the non-zero diagonal entries in $\bar{A}$. Subsequently, the minimality immediately follows by noticing that if $\mathcal{J}'$ with fewer elements than $\mathcal{J}^*$ existed, then from the proof of Theorem 4, it follows that there exists a graph partitioning with fewer partitions is possible, which would lead to a contradiction. ∎

Notice that in Theorem 5 we made two assumptions: (i) $\bar{A}$ is symmetric and $\mathcal{D}(\bar{A})$ is strongly connected, and (ii) $\bar{A}$ is zero-free diagonal. On one hand, if assumption (i) does not hold, then $\mathcal{D}(\bar{A})$ can be an arbitrary digraph. Consequently, Problem $\mathcal{P}_1$ can be reduced to a graph partitioning by considering the structural matrix $\tilde{A} = \bar{A} + \bar{A}^{\intercal}$ (i.e., we disregard directions in the state digraph). On the other hand, if assumption (ii) does not hold, then we may need to consider at least $\alpha(\bar{A}(\mathcal{G}_i))$ driving nodes for each partition $\mathcal{G}_i$, where $\bar{A}(\mathcal{G}_i)$ is the submatrix of $\bar{A}$ with columns and rows associated with the nodes in $\mathcal{G}_i$. Nonetheless, the total

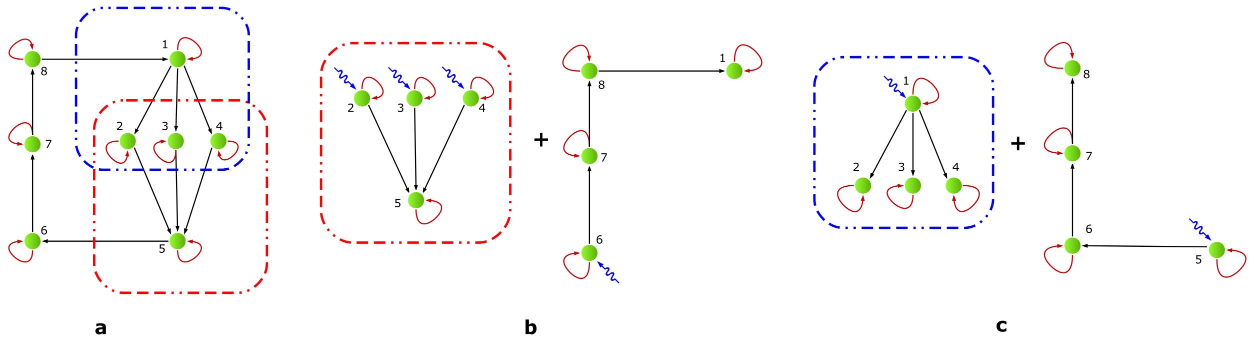

Supplementary Figure 1. (**Partition effect on digraphs.**) Example of a state digraph **(a)** that can be partitioned into two partitions with an equal number of state nodes (depicted in **(b)**-**(c)**), where each component is spanned by cycles (i.e., self-loops). The partitions depicted in **(b)** leads to a total of four dedicated driving nodes (or, equivalently, four driven nodes), whereas the partitions illustrated in **(c)** leads to a total of two dedicated driving nodes. Notice that this number of driving nodes achieves the lower bound in Theorem 3.

number of driving nodes required will depend on the specific partition (see Supplementary Figure 1).

Also, since Problem $\mathcal{P}_1$ is NP-hard, polynomial algorithms to determine the exact solution are not available. Consequently, in Algorithm 1, we propose a heuristic solution, which we refer to as *partition-based* algorithm. This algorithm follows the following two steps: (*i*) we compute a partition of the graph associated to the structural matrix $\tilde{A} = \bar{A} + \bar{A}^\intercal$ using GP methods available for undirected graphs, and (*ii*) we compute the minimum number of driving/driven nodes required to ensure structural controllability for each subgraph in the partition, by resorting to [3], [8].

ALGORITHM 1: Approximation solution to $\mathcal{P}_1$.

Input: A structural matrix $\bar{A} \in \{0, \star\}^{N\times N}$ and a structural controllability index T.

Output: An approximate solution to $\mathcal{P}_1$ given by $\tilde{\mathcal{J}}$.

1: Partition graph $\mathcal{G}$ associated with $\tilde{A} = \bar{A} + \bar{A}^\intercal$ into a collection of subgraphs $\{\mathcal{G}_i\}_{i=1}^{\lceil \frac{N}{T} \rceil}$ (using, e.g., METIS [10]);

2: If some partition $\mathcal{G}_i$ has more than T nodes, then further partition $\mathcal{G}_i$ into sub-partitions $\mathcal{G}'_1, \ldots, \mathcal{G}'_{l_i}$ with at most T nodes. Thus, the final partition can be relabelled as $\mathcal{G}''_i$ with $i = 1, \ldots, \lceil \frac{N}{T} \rceil + \gamma$, where γ denotes the total number of additional sub-partitions;

3: Compute the minimum number of driven (state) nodes, whose indices are collected in $\mathcal{J}_i^*$, that need dedicated driving nodes for each $\bar{A}(\mathcal{G}''_i)$ (following [3]), where $\bar{A}(\mathcal{G}''_i)$ is the submatrix of $\bar{A}$ with columns and rows associated with the nodes in $\mathcal{G}''_i$. Alternatively, the minimum number of driving nodes can be computed following [8];

4: Set $\tilde{\mathcal{J}} = \bigcup_{i\in\mathcal{I}} \mathcal{J}_i^*$, where $\mathcal{I} = \{1, \ldots, \lceil \frac{N}{T} \rceil + \gamma\}$.

One of the advantages of reducing our problem to that of determining the solution to GP is that its solution has to be determined using approximation (polynomial-time) algorithms with some optimality guarantees. One of the most successful software tools to solve GP problems is METIS [10]. This software package is publicly available, and has been shown to consistently lead to only $1\% - 3\%$ of partitions that do not satisfy the partitioning criteria, i.e., $|\mathcal{V}_i| \leq \left\lceil \frac{|\mathcal{V}|}{T} \right\rceil$.

Notwithstanding, one can further evaluate the quality of the solution obtained by comparing the number of nodes obtained with the lower bound in Theorem 3. In fact, we notice that, in our empirical evaluations, the total number of driving/driven nodes is often close to the lower-bound provided in Theorem 3, which implies that the solution obtained by Algorithm 1 is close to the optimal. Furthermore, the proposed partition-based algorithm achieves in practice better results then the *sequential* minimization algorithms previously suggested in the literature [13], [15] (see Supplementary Figure 2 for a brief comparison between the two).

References

[1] C. T. Chen, *Linear System Theory and Design*. Holt, Rinehart, and Winston, 1984.

[2] J.-M. Dion, C. Commault, and J. V. der Woude, “Generic properties and control of linear structured systems: a survey.” *Automatica*, pp. 1125–1144, 2003.

[3] S. Pequito, S. Kar, and A. P. Aguiar, “A framework for structural input/output and control configuration selection of large-scale systems,” *IEEE Transactions on Automatic Control*, vol. 61, no. 2, pp. 303–318, Feb 2016.

[4] M. R. Garey and D. S. Johnson, *Computers and Intractability: A Guide to the Theory of NP-Completeness*. New York, NY, USA: W. H. Freeman & Co., 1979.

[5] J. P. Hespanha, *Linear Systems Theory*. Princeton, New Jersey: Princeton Press, Sep. 2009.

[6] C. Lin, “Structural controllability,” *IEEE Transactions on Automatic Control*, no. 3, pp. 201–208, 1974.

[7] L. Markus and E. Lee, “On the existence of optimal controls,” *ASME Journal of Basic Engineering*, vol. 84, no. 1, pp. 13–20, 1962.

[8] Y.-Y. Liu, J.-J. Slotine, and A.-L. Barabási, “Controllability of complex networks,” *Nature*, vol. 473, no. 7346, pp. 167–173, May 2011.

[9] S. A. Cook, “The complexity of theorem-proving procedures,” in *Proceedings of the third annual ACM symposium on Theory of computing*, ser. STOC ’71. New York, NY, USA: ACM, 1971, pp. 151–158.

[10] G. Karypis and V. Kumar, “A fast and high quality multilevel scheme for partitioning irregular graphs,” *SIAM Journal on Scientific Computing*, vol. 20, no. 1, pp. 359–392, Dec. 1998.

[11] H. Mortazavian, “On k-controllability and k-observability of linear systems,” *Proceedings of the Fifth International Conference on Analysis and Optimization of Systems*, pp. 600–612, 1982.

[12] C. Sueur and G. Dauphin-Tanguy, “Controllability indices for structured systems,” *Linear Algebra and its Applications*, vol. 250, pp. 275 – 287, 1997.

[13] P. S. Rocha, *Output Selection for Large-Scale Structural Systems: a Matroid Theory Approach*. Department of Electrical and Computer Engineering, Faculty of Engineering, University of Porto, Porto, Portugal, 2014.

[14] S. Sundaram and C. N. Hadjicostis, “Structural controllability and observability of linear systems over finite fields with applications to multi-agent systems.” *IEEE Transactions on Automatic Control*, vol. 58, no. 1, pp. 60–73, 2013.

[15] P. Rocha, S. Pequito, S. Kar, A. Aguiar, and P. Rocha, “Sensor placement for real-time dynamic state estimation in power systems: Structural systems approach,” in *48th Asilomar Conference on Signals, Systems and Computers*, Nov 2014, pp. 1133–1137.

[16] W. Dai, Z. Gu, X. Lin, Q. S. Hua, and F. C. M. Lau, “Minimum control latency of dynamic networks,” in *2015 IEEE Conference on Computer Communications (INFOCOM)*, April 2015, pp. 648–656.

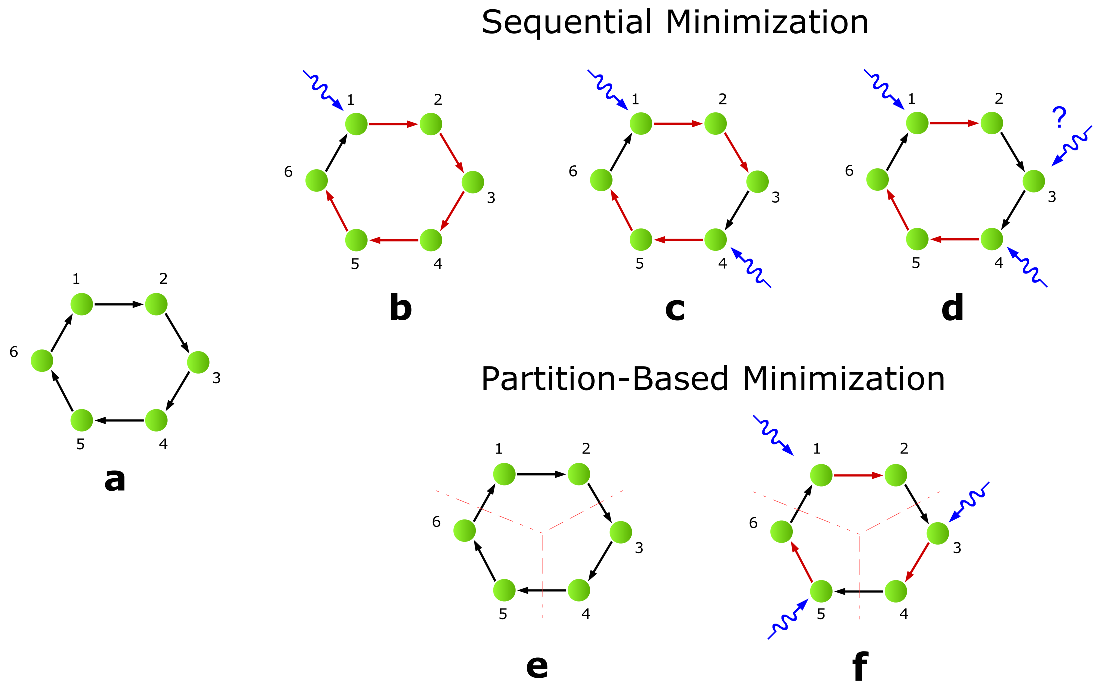

Supplementary Figure 2. (**Comparisons between algorithms to determine driving/driven nodes to ensure a given structural controllability index.**) We compare the performance of a naive (heuristic) approach, which we refer to as sequential approach, with a partition-based minimization (Algorithm 1). In particular, we apply these two approaches to find the minimum number of driven nodes to ensure a structural controllability index equal to 2 for a state digraph given by the directed cycle depicted in **(a)**. In the naive approach, we would sequentially choose a driven node to minimize the controllability index. More precisely, the first driven node can be arbitrarily assigned (e.g., node 1 in **(b)**), since the input cactus that spans the graph consists of an input stem with six state nodes. Second, we would assign a new driven node to the state node that is diametrically opposite to the state node to which the first driven node was assigned to (i.e., node 4). More precisely, we can obtain two disjoint input cacti consisting in two input stems with three state nodes each (depicted by red in **(c)**), which implies a structural controllability index equal to three. Observe that any other choice would lead to one stem having more than three state nodes, which implies a structural controllability index greater than three. Subsequently, when we consider a third driven node in any location (see **(d)**), the state digraph is spanned by three input stems, but one of these input stems still contains three state variables; hence, maintaining the same structural controllability index as in **(c)**. Nonetheless, accounting for the structure, as proposed in Algorithm 1, we can immediately obtain equal-sized partitions of the graph in **(e)**. Finally, each partition requires a driven node (as represented in **(f)**). Hence, we have three input cacti with at most two state nodes, which implies a structural controllability index equal to 2 as desired.
